# Towards Gender Harmony Dataset: Gender Beliefs and Gender Stereotypes in 62 Countries

**DOI:** 10.1038/s41597-024-03235-x

**Published:** 2024-04-17

**Authors:** Natasza Kosakowska-Berezecka, Tomasz Besta, Paweł Jurek, Michał Olech, Jurand Sobiecki, Jennifer Bosson, Joseph A. Vandello, Deborah Best, Magdalena Zawisza, Saba Safdar, Anna Włodarczyk, Magdalena Żadkowska

**Affiliations:** 1 University of Gdansk, Gdansk, Poland; 2 Medical University of Gdansk, Gdansk, Poland; 3 University of South Florida, Tampa, USA; 4 Wake Forrest University, Winston-Salem, USA; 5 Anglia Ruskin University, Cambridge, England; 6 University of Guelph, Guelph, Canada; 7 Universidad Catolica del Norte, Antofagasta, Chile

**Keywords:** Human behaviour, Culture

## Abstract

The Towards Gender Harmony (TGH) project began in September 2018 with over 160 scholars who formed an international consortium to collect data from 62 countries across six continents. Our overarching goal was to analyze contemporary perceptions of masculinity and femininity using quantitative and qualitative methods, marking a groundbreaking effort in social science research. The data collection took place between January 2018 and February 2020, and involved undergraduate students who completed a series of randomized scales and the data was collected through the SurveyMonkey or Qualtrics platforms, with paper surveys being used in rare cases. All the measures used in the project were translated into 22 languages. The dataset contains 33,313 observations and 286 variables, including contemporary measures of gendered self-views, attitudes, and stereotypes, as well as relevant demographic data. The TGH dataset, linked with accessible country-level data, provides valuable insights into the dynamics of gender relations worldwide, allowing for multilevel analyses and examination of how gendered self-views and attitudes are linked to behavioral intentions and demographic variables.

## Background & Summary

The Towards Gender Harmony project (https://towardsgenderharmony.ug.edu.pl/) started in September 2018 with more than 160 scholars who have built an international consortium that collected data in 62 countries and six continents. Our overarching goal was to analyze contemporary perceptions of masculinity and femininity using quantitative and qualitative methods, marking a groundbreaking effort in social science research. Such multinational research is important, as it helps us move beyond the WEIRD perspective of Western, Educated, Industrialized, Rich and Democratic countries which heavily predominates in psychology^1–3^.

It has been more than 30 years since a similar large cross-cultural study examined understandings of masculinity and femininity. John Williams and Deborah Best established that universally, across 26 countries, (1) communality is associated with femininity and agency is associated with masculinity, and (2) women view themselves as more communal than men and men view themselves as more agentic than women^4^. While communality and agency are universal dimensions of human evaluation^5,6^ underlying gender stereotypes and gendered self-views, the measures used in Williams and Best to capture communality and agency were not subjected to rigorous psychometric procedures for ensuring scales’ cultural invariance and equivalence. Further, because some of the data reported in Williams and Best were originally collected around 1977, they do not reflect the influence of dramatic changes in gender roles that have altered contemporary gender stereotypes^7^. It is thus important to reexamine these gender constructs today but with culturally invariant and equivalent measures. Our dataset includes contemporary data reflecting individuals’ gendered self-views, their descriptive, prescriptive, and proscriptive stereotypes about women and men, and a selection of gender beliefs and attitudes reflecting the contemporary literature of social psychology and society as a whole.

What is more, our project is unique as it examines the under-researched topic of the universality of stereotypes about men who, according to results of research (carried out so far mainly within Western cultural contexts), face strong pressures for conformity to norms such as agency, dominance, pursuit of high social status, and avoidance of femininity^8,9^. Apart from including contemporary measures of gendered self-views, attitudes, and gender stereotypes, we have also collected relevant demographic data. As a result, our Towards Gender Harmony dataset, linked with accessible country/nation-level data, offers powerful insight into the dynamics of gender relations worldwide, allowing for multilevel analyses and examination of how gender beliefs are linked to behavioral intentions and demographic variables.

This dataset has been so far used to test men’s support for gender equality across countries^10^; to establish cross-culturally valid, psychometric properties and correlates of precarious manhood beliefs^11^; to examine binary gender gaps in agentic and communal self-views^12^; to investigate whether the degree of endorsement of precarious manhood beliefs at the country level was associated with various risk-related health behaviors and outcomes^13^, to test the double standard in gender rules across countries^14^; and to test whether country-level precarious manhood beliefs were associated with more negative attitudes, fewer rights, more restrictive laws, and reduced safety for LGBTQ+ groups^15^.

## Methods

To gather data, we conducted a cross-sectional survey study employing a rigorous approach encompassing questionnaire development, data acquisition, data processing, and statistical analysis techniques. Our study aimed to investigate contemporary perceptions of masculinity and femininity across different regions of the world. We prioritize transparency and reproducibility, ensuring that our methods are accessible to fellow researchers.

### Questionnaire development

To collect pertinent information, we meticulously designed comprehensive questionnaires (refer to the Measures section for detailed content). Participants completed a battery of scales measuring a broad range of variables concerning gender beliefs and gender stereotypes (the full list is available at https://osf.io/7tza3).

### Data acquisition

We adopted the convenience sampling method, aiming to recruit a minimum of 200 participants from each country. We sent out invitations to researchers to participate in our project using mailing lists aimed at psychology researchers across the globe. These mailing lists included the International Association of Cross-Cultural Psychology, the International Academy for Intercultural Relations, and the European Association of Social Psychology. To reduce cross-national differences due to potential confounding variables (e.g., education, age) that might occur if relied on more heterogeneous samples, we asked each collaborator to obtain a university student sample of at least 100 women and men. We have also made special efforts to recruit colleagues from underrepresented countries and continents and contacted individual colleagues. Data collection occurred between January 2018 and February 2020, as part of a broader cross-cultural research project (accessible on OSF: https://osf.io/mq48y). Our participants consisted of undergraduate students who volunteered their time and, in most countries, received no compensation. We obtained ethical approval from the Ethics Board for Research projects at the Institute of Psychology, University of Gdańsk (no. 11/2018) and local Institutional Review Boards, and all participants provided informed consent. The order of measures was randomized, and data collection was facilitated through the SurveyMonkey or Qualtrics platforms. In rare instances, participants completed paper surveys.

### Data processing

We took steps to ensure data quality and integrity throughout the data processing phase. Subsequently, we conducted data cleaning procedures to identify and address missing values, outliers, and inconsistencies (detailed in the Data Records section).

By adhering to these rigorous data collection and processing procedures, we aimed to generate reliable and robust findings concerning contemporary perceptions of masculinity and femininity across diverse global contexts. This commitment to transparency and thorough methodology ensures that our research can be comprehended and replicated by other scholars in the field.

## Measures

All the measures used in the project were translated into 22 languages (Armenian, Chinese, Croatian, Danish, Dutch, English, Filipino, French, Georgian, German, Italian, Lithuanian, Norwegian, Polish, Portuguese, Romanian, Russian, Serbian, Slovak, Spanish, Turkish, Ukrainian). Bilingual scholars in psychology used the back-translation procedure to create national versions of each scale. The English version of the scales was used as the basis for all translations.

### Gendered self-views and gender stereotypes

#### Gendered self-views

Participants indicated the extent to which 12 agency-related traits, 12 communality-related traits, 12 dominance-related traits, and 12 weakness-related traits described them on a scale from 1 (does not describe me at all) to 7 (describes me well). Traits were selected from a pool of 472 prescriptive gender stereotypes (see supplementary material for the adjectives selected, Table S1 and https://osf.io/7tza3)^4,8,16^. In addition, using the same scale, they also rated the following traits: gifted in science, gifted in math, linguistically gifted, and gifted in humanities.

#### Descriptive stereotypes

Participants rated the same set of traits (12 agency-related, 12 communality-related, 12 dominance-related, 12 weakness-related) on a scale from 1 (more frequently associated with women than men) to 7 (more frequently associated with men than women). In addition, using the same scale, they also rated the following traits: gifted in science, gifted in math, linguistically gifted, and gifted in humanities.

#### Prescriptive and proscriptive stereotypes

Participants rated the prescriptive (desirable) and proscriptive (undesirable) nature of the traits (12 agency-related, 12 communality-related, 12 dominance-related, 12 weakness-related) by answering “How desirable is it in your society for a woman [man] to possess this trait?” on a scale from 1 (not at all desirable) to 7 (very desirable). In addition, using the same scale, they also rated the following traits: gifted in science, gifted in math, linguistically gifted, and gifted in humanities.

### Gender Beliefs & Attitudes

#### Precarious manhood beliefs

We administered a short version of the Precarious Manhood Beliefs (PMB) scale^17^. Based on an exploratory factor analysis of 7 items from Vandello *et al*.^17^, we selected four items with loadings >0.45 that conveyed beliefs that manhood is difficult to earn (“Some boys do not become men no matter how old they get,” “Other people often question whether a man is a ‘real man’”) and easy to lose (“It is fairly easy for a man to lose his status as a man,” “Manhood is not assured – it can be lost”). Participants indicated their agreement on a scale from 1 (strongly disagree) to 7 (strongly agree).

#### Gender essentialism

Participants’ essentialist beliefs were measured with five items (e.g., “Their underlying nature makes it difficult for men to learn to behave more like women^18^) on a scale ranging from strongly disagree (1) to strongly agree (7).

#### Ambivalent sexism

We used six items from the short version of the Ambivalent Sexism Inventory (ASI)^19^, which measures Hostile Sexism (HS) and Benevolent Sexism (BS). We selected items from Rollero *et al*.^19^ with factor loadings >0.50. HS items were: “Women seek to gain power by getting control over men,” “Women exaggerate problems they have at work,” and “When women lose to men in a fair competition, they typically complain about being discriminated against.” BS items were: “Women should be cherished and protected by men,” “Men are incomplete without women,” and “Women, compared to men, tend to have superior moral sensibility.” Items were answered on scales from 0 (strongly disagree) to 5 (strongly agree).

#### Ambivalence toward Men

We used six items from the short version of the Ambivalence toward Men Inventory (AMI)^20^, which measures Hostility toward Men (HM) and Benevolence toward Men (BM). We selected items from Rollero *et al*.^20^ with factor loadings >0.50. HM items were: “Men will always fight to have greater control in society than women,” “Men act like babies when they are sick,” and “Most men sexually harass women, even if only in subtle ways, once they are in a position of power over them.” BM items were: “Men are more willing to put themselves in danger to protect others,” “Every woman needs a male partner who will cherish her,” and “A woman will never be truly fulfilled in life if she doesn’t have a committed, long-term relationship with a man.” Items were rated on a 0 (strongly disagree) to 5 (strongly agree) scale.

#### Collective action intentions to support gender equality

To measure intention to engage in collective behaviors for gender equality, we used items taken and modified from two scales. All items were rated on a scale from 1 (not likely at all) to 7 (very likely). Instructions started with a sentence stem (“To support gender equality, how likely it is that you would …”) followed by a list of actions. Four actions, modified from Tausch *et al*.^21^, included: “participate in demonstrations”; “sign a petition”; “block buildings or streets, and “disturb events, where advocates of inequality appear.” Six actions, modified from Alisat and Reimer^22^, included: “become involved with a group (or political party) focused on gender issues/gender equality (e.g., volunteer, summer job, etc.)”; “consciously make time to be able to work on gender issues/(support) gender equality (e.g., working part time for an organization, contribute to raise awareness about gender issues, choosing activities focused on gender issues over other leisure activities)”; “participate in a community event which focused on gender issues”; “Used online tools (e.g., Instagram, YouTube, Facebook, Wikipedia, Blogs) to raise awareness about gender issues/gender equality”; “Participated in an educational event (e.g., workshop) related to gender issues/gender equality”; “Spent time working with a group/organization that deals with the connection of the gender issues/gender equality to other societal issues such as justice or inequality”.

#### Identification with gender

Participants’ identification with their gender was measured with two items (“Being a member of my gender group is an important part of how I see myself”, “To what extent you consider yourself feminine/masculine”; based on van Breen *et al*.^23^. Responses ranged from 1 (not at all) to 7 (very much).

#### Awareness of gender inequalities

Participants’ awareness of gender inequalities was measured with one item: “Overall, our society currently treats women less fairly than it treats men”. Responses ranged from 1 (strongly disagree) to 7 (strongly agree).

#### Gender roles and expectations

The items “What do you think women should prioritize?” and “What do you think men should prioritize?” were asked to assess societal attitudes and beliefs regarding gender roles. Respondents answered using a scale from 1 (Having a family) to 7 (Having a career). These items provided insights into broader societal norms related to gender roles and expectations. Individual preferences were also measured by similarly asking respondents what they would prioritize themselves – having a family or having a career.

#### Zero-sum beliefs about gender status

Participants’ zero-sum beliefs about gender status were assessed in two ways. The first was by the six-item **Zero-Sum Perspective on Gender Status** Scale (ZSPGS)^24^. The scale consists of items reflecting zero-sum beliefs in specific domains: occupational (‘More good jobs for women mean fewer good jobs for men’), power (‘The more power women gain, the less power men have’), economic (‘Women’s economic gains translate into men’s economic losses’), political (‘The more influence women have in politics, the less influence men have in politics’), social status (‘As women gain more social status, men lose social status’), and familial (‘More family-related decision making for women means less family-related decision making for men’). The second method was a more general single-item zero-sum perspective of gender status measure: ‘Declines in discrimination against women are directly related to increased discrimination against men’. Response options for each item ranged from 0 (strongly disagree) to 5 (strongly agree).

### Culture-related Relevant Measures

#### Autonomy and embeddedness values

In this study, the 10-item scale for measuring Autonomy vs. Embeddedness values was employed, following Vignoles *et al*.^25^. This scale, derived from the Portrait Values Questionnaire^26^, assessed participants’ orientations towards Autonomy (e.g., “It is important to this person to think up new ideas and be creative; to do things one’s own way.”) vs. Embeddedness (e.g., “Living in secure surroundings is important to this person; to avoid anything that might be dangerous.”) values. Participants assessed how well the description matched their own characteristics or traits from 1 (very much like me) to 6 (not at all like me).

#### Power distance beliefs

Participants’ power distance beliefs were measured using four items^27^. These items (e.g., “There should be established ranks in society with everyone occupying their rightful place regardless of whether that place is high or low in the ranking”) measured attitudes about societal ranks, requesting salary increases, questioning authority decisions, and formal communication with superiors. Responses ranged from 1 (strongly disagree) to 7 (strongly agree).

#### Subjective socio-economic status

The Subjective Social Status Ladder^28^ often referred to as the “Social Status Ladder”, was used to gauge an individual’s perception of their relative social position within their country. Respondents were asked to choose a number on the ladder from 0 (representing the lowest social status) to 10 (representing the highest social status) to indicate where they perceive themselves to be in comparison to others.

#### Attention checks

The survey also included three attention checks in which participants were asked to mark on a scale from 1 to 7 indicated numbers (“If you are reading this please choose 3”).

### Demographic variables

At the end of the questionnaire demographic information was collected. We asked participants to declare their age, study major, gender identity, education level, marital status, number of children, citizenship, and sexual orientation/identity. We also measured migration background and ethnicity (with a list of major ethnic backgrounds, if necessary adjusted/extended to meet local cultural contexts). Additionally, we ask who fulfilled the role of financial provider in the family, who fulfilled the role of homemaker in the family, and how would they describe the place they grew up (a city, a town, the countryside/remote place/rural area. Finally, our demographic part included questions about religiosity and religious denomination as well as political orientation.

## Data Records

The data comprising the TGH project results are stored in a single table. The data table is available in the repository^29^ in three formats: csv, xlsx, and Rda. The dataset contains 33,313 observations, each in a separate row, and 286 variables, each in a separate column. A detailed description of the variables can be found in the Supplementary Excel File titled ‘CodebookTGH.xlsx’, available in the Towards Gender Harmony full dataset repository^29^, which also includes a link to an interactive map with descriptive statistics and a summary of selected published statistics – the map will be developed with more analyses. The variable description consists of the following components: ‘ID’ – a unique sequential number for the item/variable (ranging from 1 to 286); ‘Variable Name’; ‘Measure’ – reference to the measurement tool used to assess this variable (containing the respective item); ‘Scale’ – the dimension, the name of the theoretical variable composed of items assigned to the scale; ‘Label’ – the content of the survey item; ‘Level of measurement’ – information about the level at which the variable/response to the item is expressed (nominal, ordinal, interval, or ratio); ‘Values’ – the range of values the variable can take; ‘Value Labels’ – possible response categories.

The dataset contains only responses provided by the study participants. Aggregated variables requiring, for example, the averaging of selected items (according to the key) must be calculated separately. To facilitate this process, we provide R code enabling the calculation of selected variables ‘TGH total scores code.R’ is available in the repository^29^.

### Sample composition

We summarize the sample composition, including sample size, gender distribution, and descriptive statistics regarding age, for 13 distinct world regions, as illustrated in Table 1. Additionally, we have provided detailed data for the 62 countries under study in the Supplementary Table 1. As previously mentioned, our participants consisted of undergraduate students who volunteered their time. After data cleaning, the final dataset comprises 33,313 observations from 62 countries across 13 world regions. As can be seen in Table 1 and Supplementary Table 1, both country-level and regional-level samples exhibit variations not only in terms of sample size but also in gender distribution and age distribution parameters.

**Table 1:** Sample Composition (Subsample Size, Gender Distribution, and Age Descriptive Statistics) across 13 World Regions.

World Region (number of countries)	N	Gender (% of Valid)					Gender - Missing data %	Age		Missing data Age %
		Female	Male	Don’t want to tell	Non-binary	Self-description		M	SD	
Africa (4)	1,530	55.91	40.93	0.34	0.74	2.08	2.75	22.34	6.04	5.49
Anglo America (2)	1,715	68.24	30.71	0.29	0.53	0.24	0.87	20.10	3.84	1.34
Central Europe (6)	4,681	59.33	39.27	0.72	0.23	0.45	5.34	25.38	8.06	6.81
East Asia (3)	1,247	64.97	32.16	0.74	1.56	0.57	2.25	20.92	4.68	7.86
Eastern Europe (8)	1,610	63.35	33.63	0.96	0.71	1.35	3.23	21.91	6.03	5.59
Latin America (7)	3,193	59.82	38.25	0.62	0.36	0.95	4.29	24.24	8.48	10.96
Middle East (4)	2,366	65.70	32.70	0.48	0.04	1.08	2.41	22.08	4.56	3.38
Northern Europe (4)	1,475	59.26	37.72	1.03	1.03	0.96	1.15	25.67	6.66	2.31
Oceania (2)	886	65.38	33.14	0.45	0.79	0.23	0.56	27.21	10.9	2.71
South Asia (5)	1,933	53.76	43.35	0.60	0.93	1.36	5.12	21.33	4.15	10.35
Southern Europe (10)	6,472	65.62	32.60	0.56	0.34	0.88	3.63	23.47	6.73	5.59
Western Europe (8)	5,361	58.75	39.54	0.49	0.70	0.51	1.87	21.89	6.30	2.50
**Total (62)**	**33,313**	**61.27**	**36.75**	**0.60**	**0.53**	**0.85**	**3.33**	**23.06**	**6.86**	**5.66**

## Technical Validation

### Data cleaning procedure

Data cleaning is a crucial preparatory step to ensure the quality and reliability of data for subsequent analysis and modeling tasks^30^. In the TGH project, the data-cleaning procedure involved the following steps:

1. Data Integration: Data from various countries were provided by collaborators in separate files. We combined data from multiple sources into a unified dataset, resolving any inconsistencies in variables or units.
2. Data Inspection: We examined the dataset to identify inconsistencies, missing values, or outliers. We paid particular attention to data integrity, making sure that values either fell within acceptable ranges or adhered to predefined rules including verification of completeness of the data in all the scales, congruity between nominal categories in different countries. During this stage, we removed records with incorrect responses to attention check questions.
3. Handling Missing Data: In the TGH database, no data imputation methods were applied. In most cases, records with missing values were retained in the database. Only observations with data gaps preventing the calculation of most measured variables were removed.
4. Outlier Treatment: Outliers were observed in the age variable. Some responses appeared to contain randomly entered numbers (e.g., 247). Observations with such responses were removed. In a few cases where birthdates were mistakenly entered as ages, we recalculated the age by subtracting the birthdate from the examination date and rounding to full years. Outliers in other variables that could potentially skew the analysis were neither removed nor adjusted.
5. Data Transformation and Scaling: Due to the use of different response scales (mainly single-item scales) in some countries compared to the standardized scale adopted for the entire study (e.g., using a scale from 0 to 6 instead of 0 to 5), linear transformations were applied to harmonize the data.
6. Data Formatting: To ensure data format consistency, some responses recorded as labels were encoded into numerical values. The mapping of labels to numbers can be found in the Supplementary Excel File titled ‘CodebookTGH.xlsx,’ available in the repository^29^.
7. Data Verification: The cleaned dataset underwent validation, including the estimation of reliability ratios for aggregated scores (see Technical Validation).

As a result of the aforementioned operations, 710 observations were removed from the initial dataset (*N* = 34,023). However, further processing is necessary, depending on the objectives of subsequent analyses and due to the presence of missing data in the dataset, to select a subset suitable for testing specific models that involve particular variables.

In addition to socio-demographic variables, the majority of variables under study are psychometric measures. As previously mentioned, the target variables are derived either by averaging/summing responses to items that make up the scale or by calculating them from the results of fitting CFA models. To assess the reliability of these measured variables, it is necessary to employ psychometric techniques. In this field, the most common method for estimating the reliability of such measurements is through the calculation of internal consistency coefficients, such as Cronbach’s (as recommended when raw scores are obtained by averaging/summing responses to items comprising a scale)^31^ or McDonald’s omega (recommended when standardized scores are to be derived using CFA results)^32^. Table 2 presents both of these reliability measures for all target variables calculated on the total sample. Detailed data on reliability coefficients calculated for each country separately are provided in Supplementary Excel File titled ‘ReliabilityTGH.xlsx’, available in the repository^29^.

**Table 2:** Reliability Measures (Cronbach’s Alpha and McDonald’s Omega) for Target Variables on the Total Sample.

Target Variable (Scale)	Number of observations (Complete cases)	Number of items	Alpha	Omega
Gender Roles and Expectations	31,973	3	0.81	0.81
Gendered Self-views (Communion)	32,063	12	0.86	0.87
Gendered Self-views (Weakness)	31,718	12	0.84	0.84
Gendered Self-views (Dominance)	31,547	12	0.84	0.84
Gendered Self-views (Agency)	32,078	12	0.88	0.88
Prescriptive Stereotypes to Women (Communion)	32,188	12	0.90	0.90
Prescriptive Stereotypes to Women (Weakness)	31,963	12	0.89	0.89
Prescriptive Stereotypes to Women (Dominance)	31,520	12	0.87	0.87
Prescriptive Stereotypes to Women (Agency)	32,041	12	0.93	0.93
Prescriptive Stereotypes to Men (Communion)	32,163	12	0.93	0.93
Prescriptive Stereotypes to Men (Weakness)	31,961	12	0.85	0.85
Prescriptive Stereotypes to Men (Dominance)	31,527	12	0.90	0.91
Prescriptive Stereotypes to Men (Agency)	32,002	12	0.91	0.92
Descriptive Stereotypes (Communion)	31,448	12	0.91	0.92
Descriptive Stereotypes (Weakness)	31,620	12	0.86	0.87
Descriptive Stereotypes (Dominance)	31,495	12	0.88	0.88
Descriptive Stereotypes (Agency)	31,986	12	0.91	0.91
Precarious Manhood Beliefs	32,723	4	0.70	0.71
Collective Action Intentions to Support Gender Equality	32,197	6	0.93	0.94
Zero-Sum Perspective of Gender Status	32,278	6	0.87	0.87
Benevolent Sexism	32,949	3	0.61	0.63
Hostile Sexism	32,543	3	0.77	0.77
Benevolence toward Men	33,018	3	0.74	0.75
Hostility toward Men	32,959	3	0.65	0.65
Gender Essentialism	32,900	3	0.75	0.75
Power Distance Beliefs	32,783	4	0.59	0.59
Autonomy Value	32,990	5	0.56	0.56
Embeddedness Value	32,958	5	0.63	0.65

As can be seen in Table 2, in the vast majority of cases, the reliability of variable measurements, as measured by the coefficient of internal consistency, exceeds the widely accepted cutoff point of >0.70^33^. Only in the case of five measures (i.e., Benevolent Sexism, Benevolence toward Men, Power Distance Beliefs, Autonomy Value, Embeddedness Value) did the results indicate reliabilities below the desired threshold. This partially can be attributed to the use of very short scales (<10 items) to measure these variables. Nevertheless it is advisable to exercise caution in interpreting the results, and it is recommended to thoroughly examine the reliability of measurements for these variables in individual countries (see Supplementary Excel File ‘ReliabilityTGH.xlsx’).

Given the cross-cultural nature of the data, it is essential to establish measurement invariance (MI) before conducting any analyses that compare results between countries. Measurement invariance refers to the consistency of a scale’s measurement properties across different groups or cultural contexts^34^. In simpler terms, it assesses whether the construct being measured is understood and interpreted in the same way across various groups or settings. Typically, researchers report three levels of measurement invariance, which are determined by parameters that are constrained to be equal across groups. The first level, configural invariance, requires the scale to demonstrate the same overall factor structure for all groups; the second level, metric invariance, necessitates that the scale items’ factor loadings be equal across the groups; and the third level, scalar invariance, demands that item intercepts be equal across groups.

For some variables in this study, such analyses have already been conducted and published^10,11,14^. These analyses involve assessing whether the measurement properties of a scale, such as factor loadings or item intercepts, remain consistent across different groups or countries. Establishing MI is crucial to ensure that any observed differences in the data result from genuine variations in the construct being measured and not from measurement bias or cultural differences.

Moreover, in the context of using the data to calculate country-level scores, it is advisable to test for psychometric isomorphism. Psychometric isomorphism extends the concept of MI by examining whether the underlying psychological structure of the measurement remains consistent across different levels, such as countries or cultures^35^. This analysis goes beyond examining the equivalence of mere measurement properties; it also investigates the constancy of the conceptual meaning and relationships among variables when considering the data at the country level.

These assessments of MI and psychometric isomorphism help ensure the validity and comparability of the data when conducting cross-cultural analyses and making country-level comparisons, providing a robust foundation for meaningful and reliable research findings.

### Supplementary information

Supplementary Table 1

## Data Availability

We provide R code enabling the calculation of selected variables. This code is available in the repository^29^ under the name TGH total scores code.R. Its proper operation requires the use of the R environment at least version 4.3.1 and the tidyverse package^36^.
