# Supplementary material for: Towards Gender Harmony Dataset: Gender Beliefs and Gender Stereotypes in 62 Countries
Source: Sci Data. 2024 Apr 17;11:392. doi: 10.1038/s41597-024-03235-x (PMC11024095; doi:10.1038/s41597-024-03235-x)
Supplement: Supplementary file 1 — Supplementary Table 1 [file 41597_2024_3235_MOESM1_ESM.docx]

**Supplementary Table**

Supplementary Table 1. Sample Composition (Subsample Size, Gender Distribution, and Age Descriptive Statistics) across 62 Countries

|  |  | **Gender (% of Valid)** | | | | | **Gender - Missing data %** | **Age** | | **Missing data Age %** |
| --- | --- | --- | --- | --- | --- | --- | --- | --- | --- | --- |
| **Country / Region** | **N** | **Female** | **Male** | **Don’t want to tell** | **Non-binary** | **Self-description** |  | **M** | **SD** |  |
| Africa |  |  |  |  |  |  |  |  |  |  |
| Ghana | 337 | 58.86 | 37.24 | 0.30 | 0.00 | 3.60 | 1.19 | 20.18 | 2.57 | 5.04 |
| Morocco | 304 | 52.51 | 45.15 | 0.67 | 0.33 | 1.34 | 1.64 | 29.05 | 9.7 | 4.93 |
| Nigeria | 468 | 54.90 | 41.61 | 0.22 | 0.87 | 2.40 | 1.92 | 21.12 | 3.14 | 4.49 |
| South Africa | 421 | 57.18 | 40.05 | 0.25 | 1.51 | 1.01 | 5.70 | 20.54 | 2.65 | 7.36 |
| Anglo America |  |  |  |  |  |  |  |  |  |  |
| Canada | 919 | 68.52 | 30.93 | 0.00 | 0.44 | 0.11 | 0.44 | 19.87 | 3.25 | 1.41 |
| USA | 796 | 67.90 | 30.45 | 0.64 | 0.64 | 0.38 | 1.38 | 20.37 | 4.41 | 1.26 |
| Central Europe |  |  |  |  |  |  |  |  |  |  |
| Czechia | 429 | 25.87 | 73.38 | 0.75 | 0.00 | 0.00 | 6.29 | 28.06 | 8.53 | 6.53 |
| Germany | 1,395 | 62.45 | 35.76 | 1.22 | 0.36 | 0.22 | 0.36 | 29.80 | 10.43 | 3.08 |
| Hungary | 773 | 80.28 | 18.87 | 0.00 | 0.14 | 0.71 | 8.08 | 22.34 | 4.27 | 8.54 |
| Poland | 845 | 55.47 | 43.20 | 0.93 | 0.40 | 0.00 | 11.24 | 22.95 | 4.67 | 10.77 |
| Slovak Republic | 648 | 50.25 | 47.57 | 0.50 | 0.00 | 1.68 | 7.87 | 21.92 | 4.58 | 9.88 |
| Switzerland | 591 | 63.88 | 35.26 | 0.34 | 0.17 | 0.34 | 0.68 | 23.54 | 5.45 | 4.57 |
| East Asia |  |  |  |  |  |  |  |  |  |  |
| China | 605 | 64.20 | 34.45 | 1.01 | 0.00 | 0.34 | 1.65 | 19.47 | 1.96 | 12.23 |
| Japan | 223 | 57.55 | 39.62 | 0.47 | 1.89 | 0.47 | 4.93 | 21.65 | 3.66 | 5.38 |
| Vietnam | 419 | 69.90 | 25.00 | 0.49 | 3.64 | 0.97 | 1.67 | 22.42 | 6.69 | 2.86 |
| Eastern Europe |  |  |  |  |  |  |  |  |  |  |
| Lithuania | 361 | 65.73 | 32.09 | 0.31 | 0.62 | 1.25 | 11.08 | 23.84 | 6.73 | 13.85 |
| Romania | 256 | 58.27 | 40.94 | 0.00 | 0.39 | 0.39 | 0.78 | 22.82 | 4.62 | 4.30 |
| Russia | 707 | 64.18 | 31.38 | 1.72 | 0.86 | 1.86 | 1.27 | 21.82 | 6.82 | 2.55 |
| Ukraine | 286 | 63.16 | 34.39 | 0.70 | 0.70 | 1.05 | 0.35 | 19.16 | 1.43 | 3.85 |
| Euroasia | 844 | 48.83 | 48.45 | 0.65 | 0.26 | 1.81 | 8.53 | 20.53 | 3.33 | 10.43 |
| Armenia | 289 | 40.79 | 56.14 | 0.00 | 0.00 | 3.07 | 21.11 | 20.02 | 1.92 | 24.22 |
| Georgia | 211 | 47.50 | 49.50 | 2.00 | 0.50 | 0.50 | 5.21 | 21.66 | 3.42 | 8.53 |
| Kazakhstan | 344 | 54.94 | 42.73 | 0.29 | 0.29 | 1.74 | 0.00 | 20.22 | 3.82 | 0.00 |
| Latin America |  |  |  |  |  |  |  |  |  |  |
| Argentina | 431 | 50.12 | 47.31 | 0.70 | 0.47 | 1.41 | 0.93 | 32.28 | 12.28 | 14.39 |
| Brazil | 1,171 | 66.42 | 31.75 | 0.37 | 0.46 | 1.01 | 6.66 | 23.99 | 7.65 | 7.60 |
| Chile | 243 | 63.25 | 34.19 | 2.56 | 0.00 | 0.00 | 3.70 | 21.70 | 5.09 | 41.56 |
| Colombia | 630 | 58.78 | 38.51 | 0.84 | 0.51 | 1.35 | 6.03 | 21.48 | 4.94 | 6.98 |
| Mexico | 344 | 52.51 | 46.31 | 0.29 | 0.29 | 0.59 | 1.45 | 23.68 | 8.92 | 13.66 |
| Suriname | 183 | 54.44 | 45.56 | 0.00 | 0.00 | 0.00 | 1.64 | 22.92 | 5.72 | 2.19 |
| Uruguay | 191 | 60.73 | 38.22 | 0.00 | 0.00 | 1.05 | 0.00 | 22.63 | 6.52 | 1.57 |
| Middle East |  |  |  |  |  |  |  |  |  |  |
| Iran | 184 | 57.95 | 40.91 | 0.57 | 0.00 | 0.57 | 4.35 | 28.95 | 8.17 | 5.98 |
| Lebanon | 135 | 69.53 | 28.91 | 1.56 | 0.00 | 0.00 | 5.19 | 19.60 | 0.85 | 4.44 |
| Turkey | 1,523 | 66.26 | 31.65 | 0.54 | 0.07 | 1.48 | 2.69 | 22.23 | 4.02 | 4.01 |
| UEA | 524 | 65.77 | 33.84 | 0.00 | 0.00 | 0.38 | 0.19 | 20.01 | 1.47 | 0.38 |
| Northern Europe |  |  |  |  |  |  |  |  |  |  |
| Denmark | 257 | 60.00 | 39.22 | 0.39 | 0.00 | 0.39 | 0.78 | 25.43 | 4.74 | 2.33 |
| Finland | 322 | 80.31 | 10.94 | 3.12 | 2.19 | 3.44 | 0.62 | 26.42 | 7.32 | 1.55 |
| Norway | 217 | 54.90 | 44.12 | 0.98 | 0.00 | 0.00 | 5.99 | 23.08 | 4.09 | 6.91 |
| Sweden | 679 | 50.37 | 47.86 | 0.29 | 1.18 | 0.29 | 0.00 | 26.19 | 7.35 | 1.18 |
| Oceania |  |  |  |  |  |  |  |  |  |  |
| Australia | 670 | 63.76 | 34.44 | 0.45 | 1.05 | 0.30 | 0.75 | 29.94 | 11.26 | 3.43 |
| New Zealand | 216 | 70.37 | 29.17 | 0.46 | 0.00 | 0.00 | 0.00 | 19.01 | 2.33 | 0.46 |
| South Asia |  |  |  |  |  |  |  |  |  |  |
| India | 396 | 60.16 | 38.24 | 0.53 | 0.27 | 0.80 | 5.56 | 22.06 | 4.99 | 7.07 |
| Indonesia | 250 | 50.22 | 44.54 | 0.00 | 0.44 | 4.80 | 8.40 | 21.02 | 3.73 | 0.00 |
| Nepal | 222 | 61.29 | 36.87 | 0.00 | 0.46 | 1.38 | 2.25 | 22.42 | 5.93 | 3.60 |
| Pakistan | 589 | 52.59 | 45.74 | 0.19 | 0.19 | 1.30 | 8.32 | 22.05 | 3.73 | 26.99 |
| Philippines | 476 | 48.31 | 47.05 | 1.69 | 2.74 | 0.21 | 0.42 | 19.78 | 2.00 | 1.05 |
| Southern Europe |  |  |  |  |  |  |  |  |  |  |
| Albania | 243 | 59.49 | 38.40 | 0.42 | 0.00 | 1.69 | 2.47 | 23.03 | 4.91 | 4.12 |
| Bosnia and Herz. | 231 | 49.75 | 48.26 | 0.00 | 0.00 | 1.99 | 12.99 | 23.01 | 5.88 | 14.29 |
| Croatia | 367 | 76.07 | 22.09 | 0.92 | 0.00 | 0.92 | 11.17 | 23.12 | 5.84 | 11.99 |
| Greece | 294 | 70.45 | 27.15 | 0.34 | 1.03 | 1.03 | 1.02 | 26.42 | 9.08 | 4.08 |
| Italy | 2,472 | 65.28 | 33.57 | 0.45 | 0.29 | 0.41 | 1.54 | 22.81 | 5.31 | 3.16 |
| Kosovo | 441 | 59.09 | 39.47 | 0.48 | 0.24 | 0.72 | 5.22 | 20.25 | 3.84 | 7.03 |
| Malta | 262 | 63.74 | 34.73 | 0.38 | 0.00 | 1.15 | 0.00 | 26.94 | 10.14 | 2.67 |
| Portugal | 174 | 80.35 | 18.50 | 0.00 | 0.58 | 0.58 | 0.57 | 22.13 | 4.90 | 1.15 |
| Serbia | 734 | 74.65 | 22.95 | 0.42 | 0.14 | 1.84 | 3.81 | 22.2 | 5.31 | 8.17 |
| Spain | 1,254 | 61.40 | 35.91 | 1.09 | 0.67 | 0.93 | 5.18 | 25.68 | 8.74 | 6.78 |
| Western Europe |  |  |  |  |  |  |  |  |  |  |
| Belgium | 1,996 | 51.12 | 47.16 | 0.36 | 0.83 | 0.52 | 3.76 | 21.6 | 6.08 | 4.61 |
| England | 755 | 58.56 | 39.30 | 0.80 | 1.20 | 0.13 | 0.93 | 22.3 | 7.53 | 0.93 |
| France | 435 | 79.17 | 17.36 | 1.62 | 0.46 | 1.39 | 0.69 | 22.28 | 6.69 | 1.38 |
| Ireland | 575 | 52.99 | 45.95 | 0.00 | 0.35 | 0.70 | 1.22 | 19.83 | 3.68 | 2.09 |
| Luxembourg | 181 | 63.48 | 34.27 | 1.12 | 1.12 | 0.00 | 1.66 | 24.61 | 5.43 | 0.00 |
| Netherlands | 899 | 66.67 | 32.66 | 0.00 | 0.34 | 0.34 | 0.56 | 20.65 | 3.40 | 0.67 |
| Northern Ireland | 307 | 60.91 | 38.11 | 0.33 | 0.00 | 0.65 | 0.00 | 22.11 | 5.57 | 0.65 |
| Wales | 213 | 61.97 | 34.74 | 1.41 | 1.41 | 0.47 | 0.00 | 30.61 | 10.42 | 4.23 |
| **Total** | **33,313** | **61.27** | **36.75** | **0.60** | **0.53** | **0.85** | **3.33** | **23.06** | **6.86** | **5.66** |
